# Esophagojejunostomy using a circular stapler vs. a linear stapler for gastric cardia cancer patients: impact of upper margin length and tumor size on the survival rate

**DOI:** 10.3389/fsurg.2024.1385754

**Published:** 2024-10-09

**Authors:** Maniragaba Dieudonne, Renguang Lv, Wenjie Xie, Qi Liu, Jianwu Jiang, Yang Fu

**Affiliations:** ^1^ First Affiliated Hospital of Zhengzhou University, Zhengzhou, Henan, China; ^2^ Gastrointestinal Surgery Department, First Affiliated Hospital of Zhengzhou University, Zhengzhou, Henan, China

**Keywords:** gastric cardia cancer, laparoscopic total gastrectomy, esophagojejunostomy, survival rate, upper margin length, tumor size

## Abstract

**Background:**

The incidence of gastric cancer is concomitantly rising with gastric cardia cancer worldwide. While the improvement of gastric cancer surgical techniques is glowing, this study assesses the impact of the upper margin length and tumor size on the survival rate for gastric cardia cancer patients who underwent total laparoscopic total gastrectomy(TLTG) or laparoscopic assisted total gastrectomy(LATG).

**Materials and methods:**

A total of 63 patients with gastric cardia cancer who underwent laparoscopic total gastrectomy were retro-prospectively collected from January 2021 to May 2023. While assessing the impact of upper margin length and tumor size on the survival rate, esophagojejunostomy using a linear stapler has been compared to a circular stapler.

**Results:**

The sixty-three patients met inclusion criteria; 32 (51%) underwent LATG and 31 (49%) underwent TLTG. Their mean age was 65 years (range, 45–77). The blood loss means in LATG and TLTG was 74.69 and 50.16 ml, respectively (*p = 0.005*), and surgery duration was higher in LATG than LATG with respective means of 247 min and 222.42 min. (*p = 0.006*). However, the tumor size means (*p = 0.5*), and upper margin length means (*p = 0.052*) were not significantly different in the LATG and TLTG groups, respectively. The number of resected and assessed lymph node was adequate in the LATG and TLTG groups. The current study still does not find an independent related risk from the upper margin length and tumor size to the survival rate according to the multiple regression analysis (*p = 0.080*).

**Conclusion:**

The upper margin length and tumor size do not have a relationship with the survival rate of the compared esophagojejunostomy (EJS) methods. The EJS using a linear stapler requires a shorter surgery duration and less blood loss than EJS using a circular stapler.

## 1 Introduction

According to the 2020 world cancer statistics, GC is still the common malignant tumor and the fourth leading cause of cancer-related death in the world. It is still the main health challenge (1–3).

Although various new drugs are being developed to treat gastric cancer, surgically curative resection remains its mainstream treatment (4, 5).

Between 1991 and 1994, Kitano et al. (6) introduced and reported the laparoscopic distal gastrectomy. A few years later, Azagra et al. (7) introduced the laparoscopic-assisted total gastrectomy. Since the development of these new methods until now, laparoscopic gastrectomy has been the method of choice for many gastrointestinal surgeons, and it has been a highly used technique in the treatment of GC patients in Eastern Asia (8).

Laparoscopic gastrectomy techniques and methods have been an important research area, and many studies have been conducted on them.

At one side, there have been reports in recent years that laparoscopic assisted total gastrectomy using a circular stapler has become more common due to the added benefits of laparoscopic extracorporeally technical facilities and safety. The laparoscopic assisted total gastrectomy (LATG) needs a mini-laparotomy for reconstruction with use of CS(circular stapler) devices which cannot pass through the trocar.

At another side they reported that an intracorporeal reconstruction with use of linear stapler is a technique mostly used to perform a totally laparoscopic total gastrectomy (TLTG) (5, 9). In comparison to a circular stapler, a linear stapler offers several benefits: it can be executed intracorporeally, negating the need for an assistant incision; it results in reduced blood loss and complications such as anastomosis stricture and leakage; it is simple to execute under direct laparoscopic vision; and it is suitable for high-level esophagojejunostomy (10). The surgical short- and long-term results of intracorporeal and extracorporeal esophagojejunostomies during laparoscopic total gastrectomy have been compared in numerous studies (11). Therefore, the goal of the current study comparing esophagojejunostomy (EJS) using a LS and EJS using a CS in gastric cardia cancer patients was assessing an impact of upper margin length and tumor size on the survival rate in general.

## 2 Materials and methods

We retroprospectively collected data on 63 gastric cardia cancer patients who underwent laparoscopic total gastrectomy from the same highly experienced team of surgeons. This study was retrospective from January 2021 to November 2022 and prospective from December 2022 to May 2023. The current study was conducted in the gastrointestinal surgery department of the first affiliated hospital of Zhengzhou University and covered the period of 29 months. Patients were divided into two groups according to the use of a linear or circular stapler.

The first group of 32 patients underwent laparoscopic assisted total gastrectomy (LATG), and the second group of 31 patients underwent total laparoscopic total gastrectomy (TLTG).

### 2.1 Inclusion criteria

Were included in our study, the patients who underwent a totally laparoscopic total gastrectomy, and patients who underwent a laparoscopic assisted total gastrectomy for gastric cardia cancer (GCC) during the current study period.

### 2.2 Exclusion criteria

**Figure 1:**
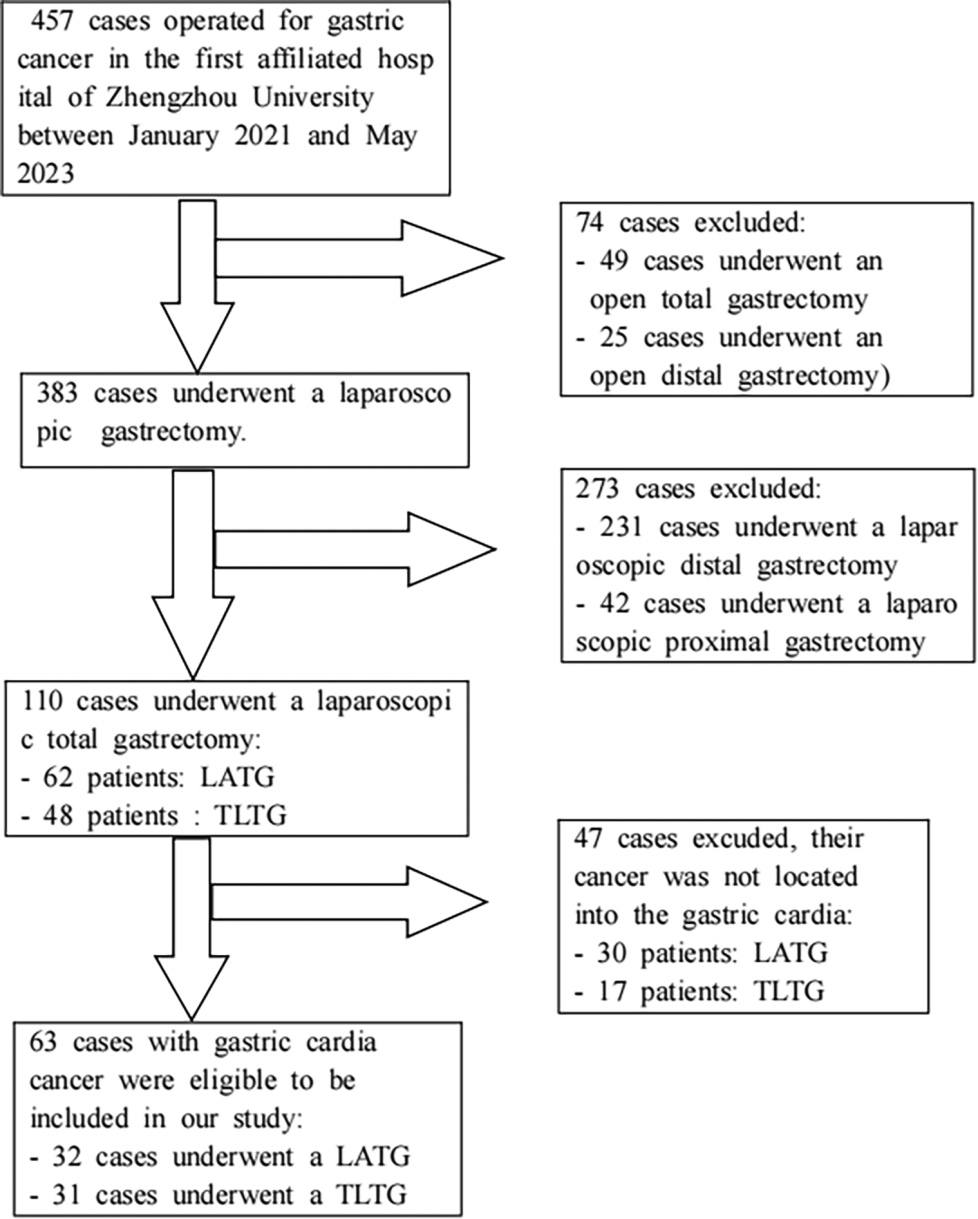
Process of screening and selection of patients.

As mentioned in the figure below (Figure 1), were excluded from our study, patients who didn’t meet the inclusion criteria:

- All patients who underwent open total gastrectomy for gastric cardia cancer were excluded from our study.
- All patients who underwent surgery for gastric cancer with tumor not located into gastric cardia were excluded.

### 2.3 Datacollection and statistical analysis

Our data have been collected according to variables such as patient characteristics (gender, age, body mass index, comorbidities, history of abdominal surgery, neoadjuvant chemotherapy, and tumor location). Perioperative baselines (surgery duration, blood loss, intraoperative complications). Postoperative baselines (pathological findings: upper margin length, tumor size, upper margin invasion, pathological tumor stage, pathological node stage, number of lymph nodes resected, number of positive lymph nodes, histological type). Other postoperative baselines: *Postoperative complications (anastomosis leakage, anastomotic stricture, surgical wound infection, etc.) *Postoperative hospital stay and survival rate (alive, recurrence, or death). The patient’ phone number and the patient's attendant's phone number had been used to follow up. The follow-up schedule has been established as follows: for the retrospective part, patients or patients’ families were contacted using phone call once, and data for routine follow-up were found in the hospital data base. For the prospective part, after surgery, patients have been evaluated at 3 months and 6 months following surgery.

The below surgical technique description explains clearly the surgical procedures used during surgeries:

### 2.4 Description of surgical procedure

#### 2.4.1 Positioning and disinfection

Patient under general anesthesia in supine and reverse Trendelenburg with open-legs position. After disinfection of the abdomen and coverage by sterilized drapes, the operator stood to the left side, assistant to the right side, and the laparoscopist between the two legs.

#### 2.4.2 Port placement and general exploration

In total, five trocars are necessary and placed through the small surgical holes made in the abdomen. Through the skin incision at 1 cm below the umbilicus, a 12 mm trocar is entered into the abdomen. After pneumoperitoneum with carbon dioxide is established, a general inspection of the peritoneal cavity is done. The other four trocars were entered into the abdominal cavity under endoscopic vision.

#### 2.4.3 Omentum and ligaments division, lymphadenectomy, blood vessel breaking and ligation

**Figure 2:**
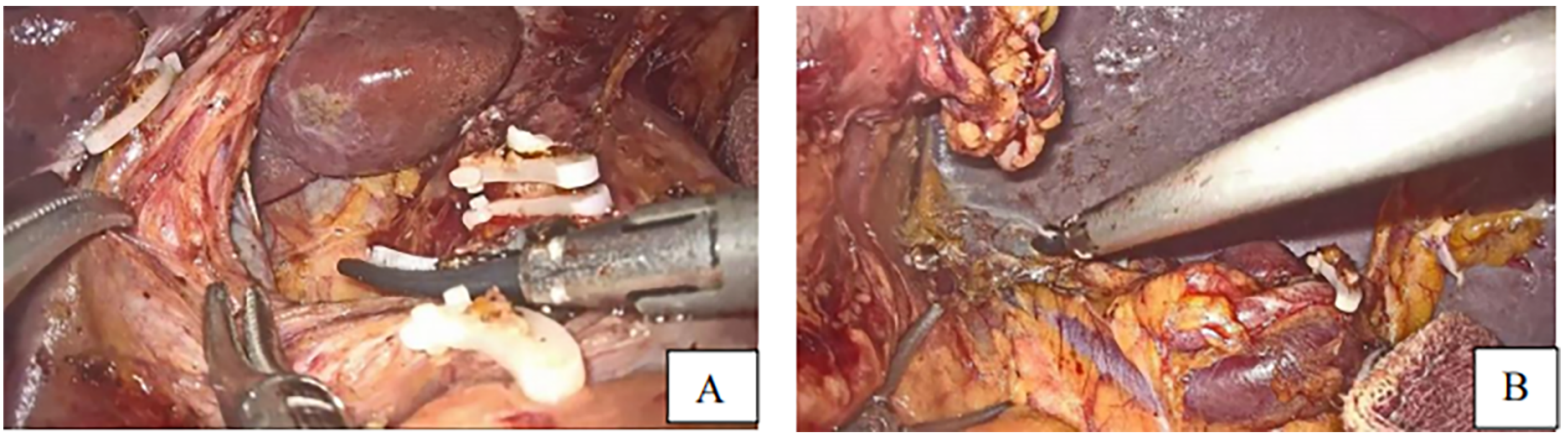
**(A,B)** standard D2 lymphadenectomy performed.

**Figure 3:**
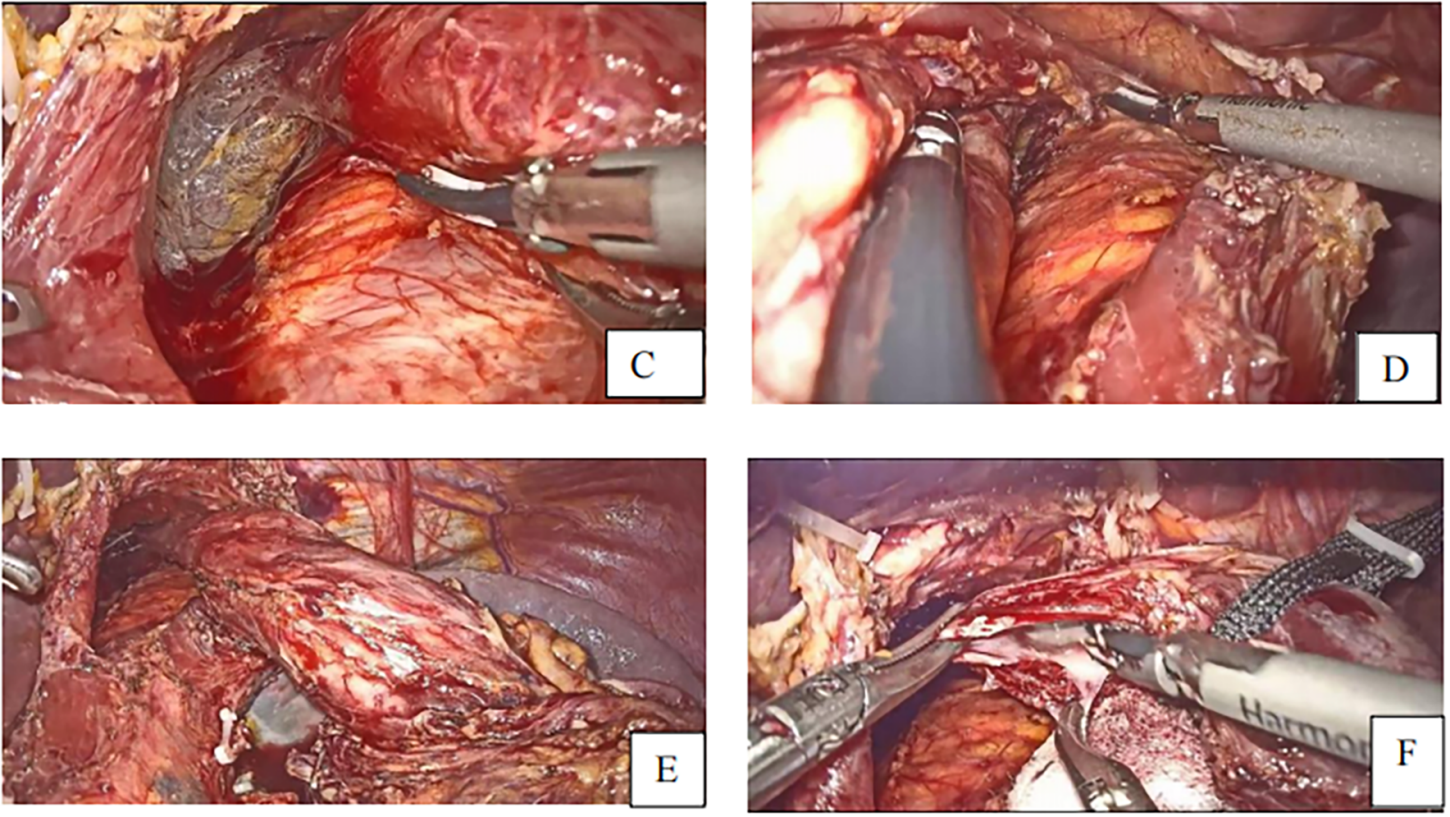
**(C–E)** fully free the lower esophagus with a 5-7 cm **(F)** incision of esophagus to make a small opening with harmonic scalpel.

After division of the greater omentum with the transverse colon, ligament division, lymph node dissection (Figures 2A,B) starting by the dissection of the 4th group of lymph nodes, 5th, 7th, 8th, 9th, 12th, 3rd, 1st, and eleventh group of lymph nodes, vessel breaking, and ligation, the duodenum was dissected following gastric mobilization. Hiatus dissection and finally esophagus mobilization (Figures 3C–E).

### 2.5 Esophagojejunal anastomosis using a linear stapler

After esophageal mobilization, the stomach was pulled up using the line tractor, and then the esophageal small opening was made at the posterolateral side using the harmonic scalpel (Figure 3F).

### 2.6 Preparation of the jejunal limb

**Figure 4:**
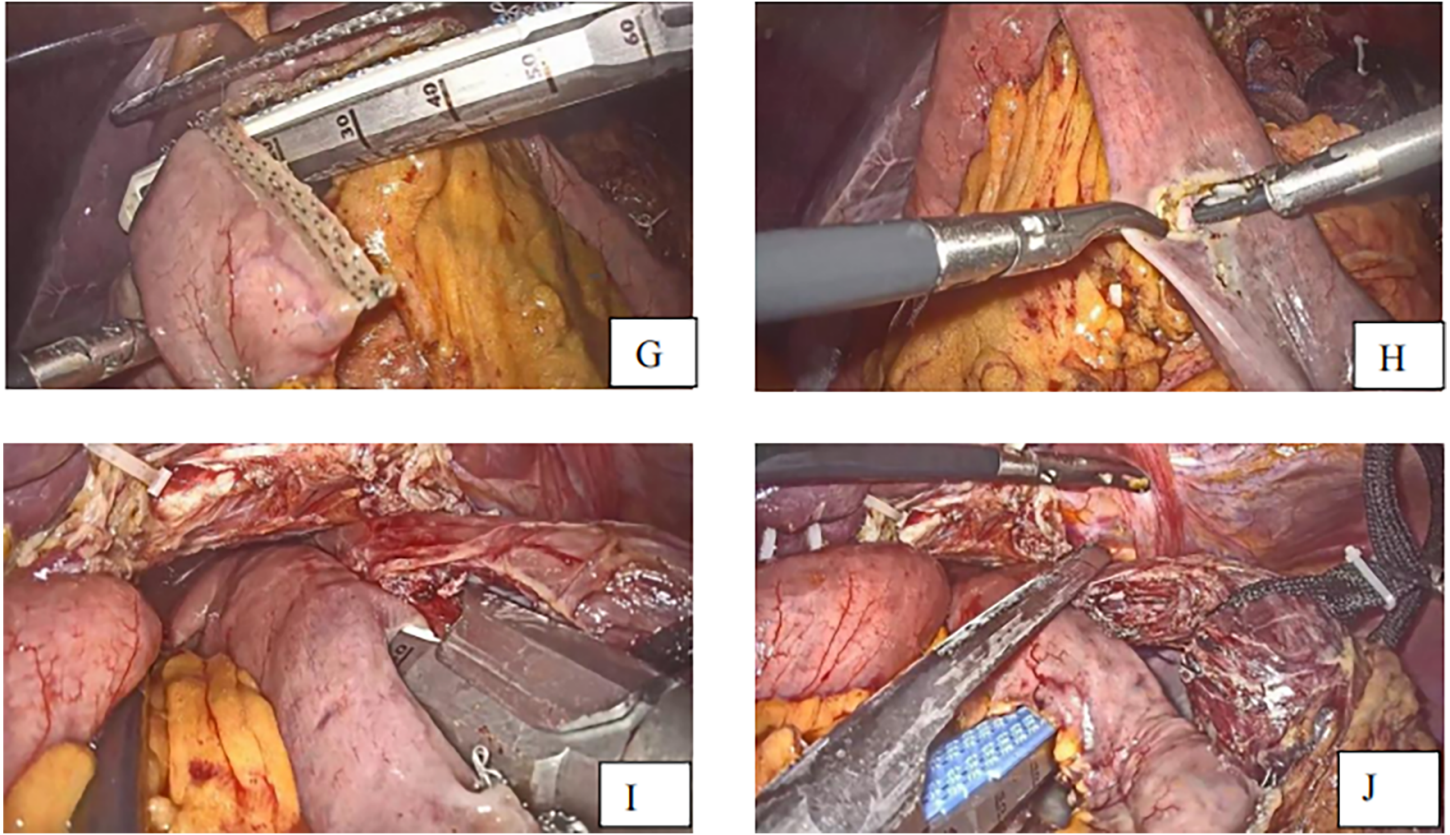
**(G)** Transection of small intestine **(H)** small opening to the distal jejunal stump **(I)**. Linear staplers anastomose the esophagus and jejunum **(J)**. Closure of the common hole and transection of specimen to the anastomosis.

After identifying the vessels’ structure in the mesentery, the jejunum is transected at approximately 20–30 cm distal from the Treits's ligament using a linear stapler. A small entry for stapler insertion is created at about 5 cm from the distal jejunal stump (Figures 4G,H).

### 2.7 Esophagojejunal stapling

**Figure 5:**
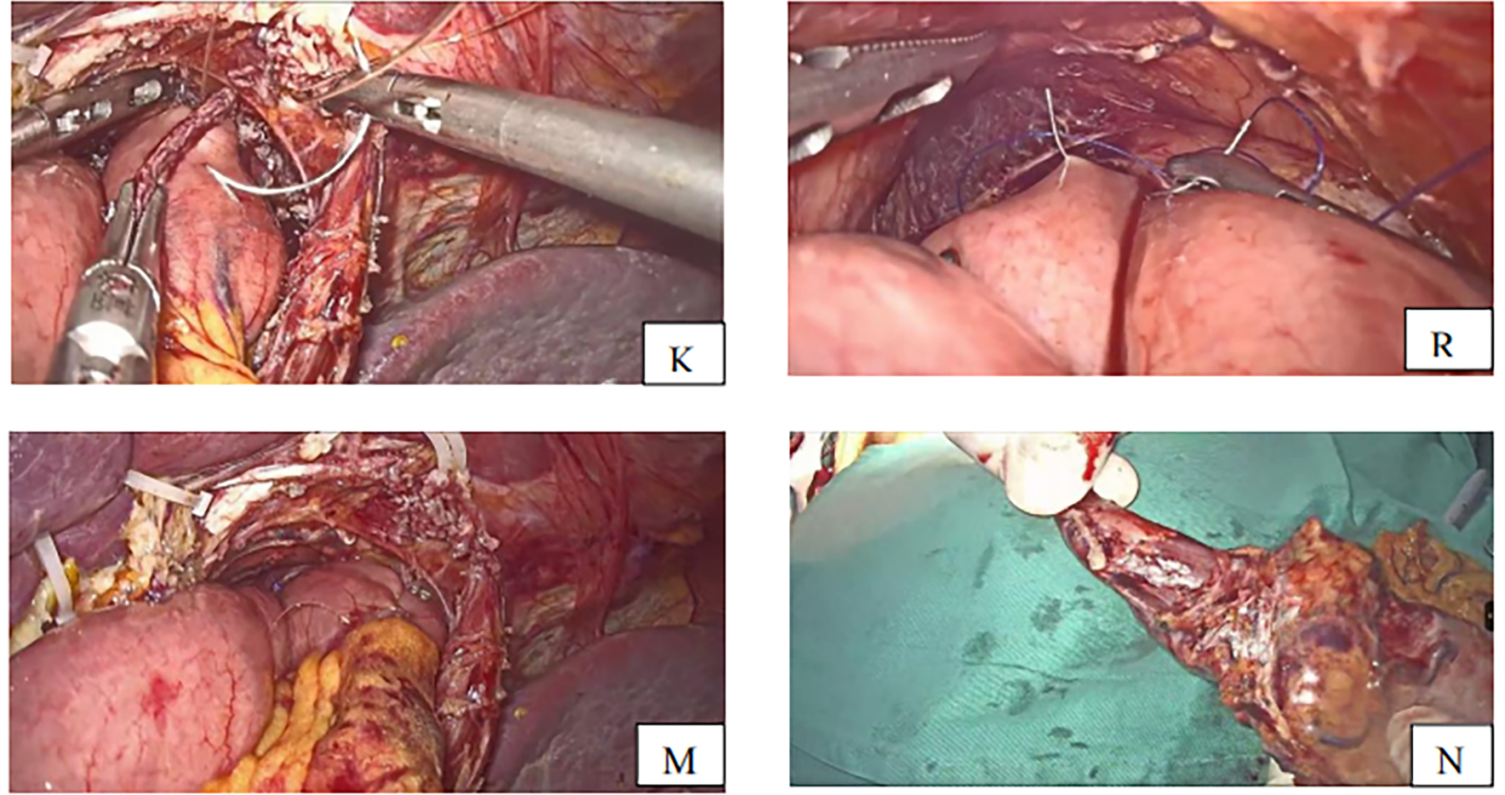
**(K)** Anastomotic tail sutured to prevent bleeding **(R)** anastomosis end is sutured with one stitch to decompress. **(M)** Anastomosis completed **(N)** specimen removed with esophagus about 3 cm.

The semi-separated staple is introduced from the jejunal hole, and the jejunal limb is subsequently extended to the esophagus, where the small hole was created. The stapler is moved to the posterior site of the esophagus via the esophageal hole, and the esophagojejunostomy is carried out after firing the linear stapler. The common entry hole was closed using another linear stapler introduced through the port on the right side, which separated the specimen and the anastomosis at 0.5 cm from the esophagojejunal anastomosis (Figures 4I,J). The anastomosis end and anastomotic tail were sutured to reduce pressure and prevent bleeding (Figures 5K,R,M).

### 2.8 Jejuno-jejunostomy

After completing the esophagojejunal anastomosis, the specimen was removed (Figure 5N) through a small incision of about 5 cm made in the upper midline, and then a side-to-side jejuno-jejunostomy was performed using a linear stapler. The length of the jejunal pouch from the esophagojejunal anastomosis was about 40 cm. The mesentery gap was closed to avoid Petern's hernia.

### 2.9 Esophagojejunal anastomosis using a circular stapler

After lymph node dissection, stomach and esophagus mobilization, the pneumoperitoneum was stopped, and a 7–10 cm mini-laparotomy was created in the upper midline. The stomach was pulled up by the operator, and the purse-string suture was located about 1 cm from the incision line, which was decided in advance. The specimen was separated from the esophagus and removed. The stapler nail seat was placed in the esophageal stump and maintained by tightening knot of the purse line.

### 2.10 Preparation of the jejunal limb

The small intestine was pulled out through the mini-laparotomy incision, the distal and proximal ends were identified, and it was then cut at about 30 cm from the Treits’s ligament.

### 2.11 Esophagojejunostomy

The circular stapler was inserted in the jejunum via the distal jejunal stump and then lifted anteriorly through the colon and connected to the esophageal stapler nail seat. The esophagojejunostomy with a circular stapler was completed after the circular stapler was heated. The jejunal stump was closed by an endoscopic stapler. The end-to-end esophagojejunostomy anastomosis is reinforced by hand-sewn knots to avoid anastomotic leakage.

### 2.12 Jejunojejunal anastomosis with a circular stapler

After carrying out the esophagojejunostomy with a circular stapler, the circular stapler nail seat was inserted in the jejunum via a small opening made on the antimesenteric side of the jejunum at about 50 cm from the esophagojejunal anastomosis. A circular stapler introduced via the proximal jejunal stump was connected to the stapler nail seat, and the end-to-side jejujojejunal anastomosis was finished. The anastomosis was reinforced by hand-sewn stitches.

Process of screening and selection of patients (Figure 1).

## 3 Statistical analysis

The statistical analysis was performed using SPSS software for windows, and statistical analysis in R version 4.3.1 was used. The Fisher's exact test, Welch two-sample *t*-test, and Wilcoxon rank sum test were used. A *p*-value <005 was considered statistically significant.

## 4 Results

From January 2021 to May 2023, we retroprospectively conducted a study on EJS in totally laparoscopic total gastrectomy vs. EJS in laparoscopic assisted total gastrectomy for gastric cardia cancer patients. This study aimed to assess the impact of upper margin length and tumor size on the survival rate. Patient characteristics such as age, gender, BMI, comorbidities, history of previous abdominal surgery, neoadjuvant chemotherapy, upper margin length, tumor size, blood loss, mobilized esophagus, surgery duration, resected lymph nodes, positive lymph nodes, intraoperative complications, pathological tumor category, pathological node category, histology type, margin invasion, postoperative hospital stay, and survival rate have been represented in the tables below:

The results in the (Table 1) show that among the 63 patients who underwent LTG, 52.0 (82.5%) remained alive, and 11.0 (17.5%) developed a recurrence or died. However, the results presented in the (Table 2) show that among the 32 patients in the LATG group, 25 (78%) remained alive, 6 (19%) have died, and 1 (3%) have developed recurrence. While among the 31 patients in the TLTG group, 27 (87.1%) remained alive, 3 (9.7%) have died, and 1 (3.2%) has developed a recurrence.

**Table 1:** Distribution of patients according the outcome after surgery.

Characteristic	*N* = 63^a^	95% CI^b^
Survival rate		
Alive	52.0 (82.5%)	70%, 91%
Died or recurrence	11.0 (17.5%)	9.4%, 30%

^a^ Frequency (%).

^b^ CI, confidence interval.

**Table 2:** Distribution of patients according to the survival rate in LATG and TLTG groups.

Characteristic	Alive, *N* = 52^a^	Died, *N* = 9^a^	Recurrence, *N* = 2^a^	Overall, *N* = 63^b^	*p*-value^c^
Group					0.7
LATG	25 (78%)	6 (19%)	1 (3%)	32	
TLTG	27 (87.1%)	3 (9.7%)	1 (3.2%)	31	
Overall, *N* = 63^2^	52 (82.5%)	9 (14.3%)	2 (3.2%)	63	

^a^ *n* (%).

^b^ *n*.

^c^ Fisher's exact test.

## 5 Bivariate statistical analysis

The results presented in the (Table 3) show that gender, comorbidities, neoadjuvant chemotherapy, and age do not have a statistically significant influence on the outcome (survival rate) of our patients. However, the BMI has a statistically and significantly influence on the survival rate (*p = 0.012*).

**Table 3:** Preoperative baselines and survival rate linkage.

Characteristic	Alive, *N* = 52^a^	Died or recurrence, *N* = 11^a^	*p*-value^b^
Gender			0.13
Female	68.8%	31.2%	
Male	87.2%	12.8%	
Comorbidities			0.054
Coronary heart disease	0.0%	100.0%	
Coronary heart disease and Treponema pallidium disease	0.0%	100.0%	
Diabetes and high blood pressure	83.3%	16.7%	
Diabetes, high blood pressure and cerebral infarction	50.0%	50.0%	
Hepatitis B and coronary heart disease	100.0%	0.0%	
High blood pressure	75.0%	25.0%	
None	87.5%	12.5%	
NACT			>0.9
Neoadjuvant chemotherapy	83.3%	16.7%	
None	82.2%	17.8%	
History of surgery			0.3
Appendectomy	100.0%	0.0%	
Liver lobectomy	0.0%	100.0%	
None	83.1%	16.9%	
Pancreaticoduodenobile surgery	100.0%	0.0%	
Age	63.98	67.64	0.080
BMI (Kg/m^2^)	23.89	22.47	0.012

^a^ %; mean.

^b^ Fisher's exact test; Welch two sample *t*-test.

The results presented in the (Table 4) show the intraoperative characteristics, such as mobilized esophagus, surgery duration, and blood loss. They do not statistically influence the survival rate. Their *p*-values close *0.05*. In the (Table 5), the pathology tumor category, the pathology node category, and the upper margin invasion have statistically significant influence on the survival rate, with respective *p*-values of *0.57, 0.022*, and *0.028* However, the upper margin length, tumor size, ratio of upper margin length and tumor size, resected LNs, and positive LNs have a *P*-value higher than 0.05.

**Table 4:** Intraoperative baselines and survival rate linkage analysis.

Characteristic	Alive, *N* = 52^a^	Died or recurrence, *N* = 11^a^	*p*-value^b^
Intraoperative complication			0.2
Bleeding	0.0%	100.0%	
None	83.9%	16.1%	
Mobilized esophagus (cm)	5.29	5.18	0.5
Surgery duration	233.31	242.45	0.4
Blood loss (ml)	58.94	80.00	0.3

^a^ %; mean.

^b^ Fisher's exact test; Welch two sample *t*-test; Wilcoxon rank sum test.

**Table 5:** Crossover of postoperative pathological baselines and survival rate.

Characteristic	Alive, *N* = 52^a^	Died or recurrence, *N* = 11^a^	*p*-value^b^
Pathological tumor category			0.057
T1	80.0%	20.0%	
T2	100.0%	0.0%	
T3	80.0%	20.0%	
T4	0.0%	100.0%	
Pathological node category			0.022
N0	92.9%	7.1%	
N1	87.5%	12.5%	
N2	71.4%	28.6%	
N3	40.0%	60.0%	
Histology type			>0.9
Highly differentiated adenocarcinoma	100.0%	0.0%	
Moderately differentiated adenocarcinoma	82.4%	17.6%	
Poorly differentiated adenocarcinoma	77.8%	22.2%	
Well differentiated adenocarcinoma	85.2%	14.8%	
Upper margin status			0.028
No invasion	85.2%	14.8%	
Upper margin invasion	0.0%	100.0%	
Upper margin length	2.67	2.73	0.7
Tumor size	4.0	4.50	0.2
Ratio (tumor size/upper margin length)	1.52	1.80	0.15
Lymph nodes resected	20.96	23.18	0.2
Positive lymph nodes	1.65	4.91	0.2

^a^ %; Mean.

^b^ Fisher's exact test; Wilcoxon rank sum test.

According to the results presented in the (Table 6), postoperative complications and the postoperative hospital stay do not have a statistically significant influence on the survival rate.

**Table 6:** Crossover postoperative complications and survival rate.

Characteristic	Alive, *N* = 52^a^	Died or recurrence, *N* = 11^a^	*p*-value^b^
Post-operative Complication			0.2
Anastomotic leakage	100.00%	0.00%	
Surgical wound infection	0.00%	100.00%	
None	83.10%	16.90%	
Post-operative hospital stay	11.08	11.27	0.2

^a^ %; mean.

^b^ Fisher's exact test; Wilcoxon rank sum test.

## 6 Logistic regression for evaluation of impact of upper margin length and tumor size on the survival rate

The results presented in the (Table 7) are from multiple logistic regression, and we find that the upper margin length and tumor size do not influence the survival rate. Indeed, their respective associated *p*-values (0.7 and 0.2) are well above the significance threshold (0.05). However, we also find that the survival rate is explained only by BMI, the pathology tumor category, the pathology node category, the lymph node category, the positive lymph node category, the upper margin invasion, and the age category at the threshold of 5%. Characteristics such as ratio (tumor size/upper margin length) and comorbidities significantly explain survival rate, respectively, with thresholds of 8% and 7%.

**Table 7:** Multiple logistic regression.

Characteristic	*N*	OR^a^	95% CI^a^	*p*-value
Gender	63			0.11
Female		—	—	
Male		0.32	0.08, 1.30	
BMI (kg/m^2^)	63	0.69	0.46, 0.97	0.033
NACT	63			>0.9
Neoadjuvant chemotherapy		—	—	
None		1.08	0.27, 5.44	
Upper margin length category	63			0.7
2 thru 3		—	—	
3 thru highest		0.64	0.03, 4.23	
Tumor size (cm)	63	1.46	0.83, 2.78	0.2
Ratio (tumor size/upper margin length)	63	3.62	0.87, 23.9	0.080
Mobilized esophagus (cm)	63	0.77	0.24, 2.08	0.6
Operative duration (min)	63	1.01	0.99, 1.03	0.4
Pathological tumor category	63			0.040
T1		—	—	
T2		0.00		
T3		1.00	0.13, 20.8	
T4		462,595,173	0.00, NA	
Pathological node category	63			0.038
N0		—	—	
N1		1.86	0.20, 16.9	
N2		5.20	0.88, 42.0	
N3		19.5	2.16, 253	
Histology of type	63			0.9
Highly differentiated adenocarcinoma		—	—	
Moderately differentiated adenocarcinoma		3,353,863	0.00, NA	
Poorly differentiated adenocarcinoma		4,471,817	0.00, NA	
Well differentiated adenocarcinoma		2,721,976	0.00, NA	
Margin status	63			0.007
None		—	—	
Upper margin invasion		90,430,085	0.00, NA	
Lymph nodes category	63			0.019
Lowest thru 25.5		—	—	
25.5 thru 26.5		12.6	1.07, 293	
26.5 thru 30.5		0.00		
30.5 thru Highest		12.6	1.07, 293	
Positive lymph nodes category	63			0.024
Lowest thru 3.5		—	—	
3.5 thru highest		5.36	1.26, 23.1	
Age category	63			0.007
Lowest thru 62.5		—	—	
62.5 thru 71.5		7.67	1.22, 150	
71.5 thru 72.5		359,981,298	0.00, NA	
72.5 thru highest		2.88	0.11, 78.7	
Blood loss category	63			0.059
Lowest thru 185		—	—	
185 thru highest		81,387,076	0.00, NA	
Post-operative hospital stay category	63			0.081
Lowest thru 17		—	—	
17 thru 20		204,215,099	0.00, NA	
20 thru highest		0.00		
Comorbidities combined	63			0.079
Yes		—	—	
No		0.29	0.07, 1.16	
History of surgery	63			0.7
Yes		—	—	
No		0.61	0.07, 13.1	
Post-operative complication	63			0.7
Yes		—	—	
No		0.61	0.07, 13.1	

^a^ OR, odds ratio; CI, confidence interval.

The preoperative baselines of the LATG and TLTG groups are presented in the (Table 8). The LATG and TLTG groups consisted of 32 and 31 patients, respectively. The mean age was 63.69 and 65.58, respectively (*p = 0.3*), without a statistically significant difference. The male sex was represented at 81.3% and 61.7%, and female sex was represented at 18.8% and 32.3% in the LATG and TLTG groups. The frequency of comorbidities was 29.1% and 19.4% in the LATG and TLTG groups, respectively. A frequency of 21.9 and 35.5% in the LATG and TLTG groups were under NAC before surgery. The frequency of previous abdominal surgery was 9.4% and 3.2% (*p > 0.5*) in the LATG and TLTG groups, respectively. The BMI mean was 23.49 and 23.79 (*p = 0.6)* in the LATG and TLTG groups, respectively.

**Table 8:** Comparison of preoperative baselines in LATG and TLTG groups.

Characteristic	LATG, *N* = 32^a^	TLTG, *N* = 31^a^	Difference^b^	95% CI^b^^,^^c^	*p*-value^b^
Gender			0.31	−0.18, 0.81	
Female	18.8%	32.3%			
Male	81.2%	67.7%			
Comorbidities			0.59	0.09, 1.1	
Coronary heart disease	0.0%	3.2%			
Coronary heart disease and treponema pallidium disease	3%	0.0%			
Diabetes and high blood Pressure	9.4%	9.7%			
Diabetes, high blood pressure and cerebral infarction	6.3%	0.0%			
Hepatitis B and coronary heart disease	3.1%	0.0%			
High blood pressure	6.3%	6.5%			
None	71.9%	80.6%			
NACT			0.30	−0.19, 0.80	
Neoadjuvant chemotherapy	21.9%	35.5%			
None	78.1%	64.5%			
History of surgery			0.37	−0.13, 0.86	
Appendectomy	3.1%	3.2%			
Liver lobectomy	3.1%	0.0%			
Pancreaticoduodenobile surgery	3.1%	0.0%			
None	90.7%	96.8%			
Age	63.69	65.58	−1.9	−5.5, 1.7	0.3
BMI (Kg/m^2^)	23.49	23.79	−0.30	−1.3, 0.75	0.6

^a^ %; Mean.

^b^ Standardized mean difference; Welch two sample *t*-test.

^c^ CI, confidence interval.

The results in the (Table 9) are significantly and statistically different between the two groups in terms of operative duration and blood loss and their respective *P*-values (0.006 and 0.005). However, intraoperative complications and mobilized esophagus are not statistically or significantly different between the two groups.

**Table 9:** Comparison of intraoperative baselines between LATG and TLTG groups.

Characteristic	LATG, *N* = 32^a^	TLTG, *N* = 31^a^	Difference^b^	95% CI^b^^,^^c^	*p*-value^b^
Intra-operative Complication			0.25	−0.24, 0.75	
Bleeding	3.1%	0.0%			
None	96.9%	100.0%			
Mobilized esophagus (cm)	5.22	5.32	−0.10	−0.43, 0.23	0.5
Operative duration (min)	247.00	222.42	25	7.4, 42	0.006
Blood loss (ml)	74.69	50.16	25	7.8, 41	0.005

^a^ %; mean.

^b^ Standardized mean difference; Welch two sample *t*-test.

^c^ CI, confidence interval.

The Table 10 presents the results of postoperative pathological baselines. The mean number of resected lymph nodes was 19.50 and 25.00, with a *p = 0.3* in the LATG and TLTG groups, respectively. The mean positive lymph nodes were 0.00 (0, 2) and 1.00 (0, 2) (*p = 0.4*) in the LATG and TLTG groups, respectively. For the two surgical methods, the number of resected and assessed lymph nodes is not significantly different.

**Table 10:** Comparison of postoperative pathological baselines between LATG and TLTG groups.

Characteristic	LATG, *N* = 32^a^	TLTG, *N* = 31^a^	Difference^b^	95% CI^b^^,^^c^	*p*-value^b^
Pathological tumor category			0.34	−0.16, 0.83	
T1	3.0 (9.4%)	2.0 (6.5%)			
T2	7.0 (21.9%)	5.0 (16.1%)			
T3	21.0 (65.6%)	24.0 (77.4%)			
T4	1.0 (3.1%)	0.0 (0.0%)			
Pathological nodes category			0.29	−0.21, 0.79	
N0	16.0 (50.0%)	12.0 (38.7%)			
N1	7.0 (21.8%)	9.0 (29.0%)			
N2	6.0 (18.8%)	8.0 (25.8%)			
N3	3.0 (9.4%)	2.0 (6.5%)			
Histological type			1.3	0.77, 1.9	
Highly differentiated adenocarcinoma	0.0 (0.0%)	1.0 (3.2%)			
Moderately differentiated adenocarcinoma	3.0 (9.4%)	14.0 (45.2%)			
Poorly differentiated adenocarcinoma	16.0 (50.0%)	2.0 (6.4%)			
Well differentiated adenocarcinoma	13.0 (40.6%)	14.0 (45.2%)			
Upper margin status			0.01	−0.49, 0.50	
None	31.0 (96.9%)	30.0 (96.8%)			
Upper margin invasion	1.0 (3.1%)	1.0 (3.2%)			
Upper margin length (cm)	3.00 (2.00, 3.00)	2.00 (2.00, 3.00)	0.33	0.00, 0.67	0.052
Tumor size (cm)	4.00 (3.75, 5.00)	4.00 (4.00, 5.00)	−0.23	−0.83, 0.38	0.5
Ratio(tumor size/upper margin length)	1.30 (1.00, 2.00)	2.00 (1.00, 2.00)	−0.22	−0.48, 0.04	0.092
Lymph nodes resected	19.50 (18.00, 22.00)	22.00 (19.00, 25.00)	−1.2	−3.4, 1.1	0.3
Positive lymph nodes	0.00 (0.00, 2.00)	1.00 (0.00, 3.00)	1.0	−1.2, 3.2	0.4

^a^ *n* (%); median (IQR).

^b^ Standardized mean difference; Welch two sample *t*-test.

^c^ CI, confidence interval.

The mean tumor sizes were 4 cm (3.75–5) and 4 cm (4–5) (*p* = *0.5*) into laparoscopic assisted total gastrectomy and totally laparoscopic total gastrectomy groups, respectively. The mean upper margin length was 3 cm (2–3) and 2 cm (2–3) (*p* =* 0.052*) in the laparoscopic assisted total gastrectomy (LATG) and total laparoscopic total gastrectomy(TLTG) groups, respectively. The mean ratios (tumor size/upper margin length) were 1.3 (1–2) and 2 (1–2) (*p = 0.092*) into the laparoscopic assisted total gastrectomy(LATG) and total laparoscopic total gastrectomy(TLTG) groups, respectively. One patient (3.1%) in LATG and one patient (3.2%) in TLTG presented an upper margin invasion. Frequencies for histological types were highly differentiated to a rate of 3.2% in the TLTG group, moderately differentiated to 9.4% and 45.2% in the LATG and TLTG groups, respectively. Well differentiated to 40.6% and 45.2% into the laparoscopic assisted total gastrectomy(LATG) and total laparoscopic total gastrectomy(TLTG) groups, respectively. Poorly differentiated to 50% and 6.4% in the LATG and TLTG groups, respectively. The pT category is pT1 (9.4% and 6.5%) in the two groups(LATG,TLTG), respectively. pT2 (21.9% and 16.1%), pT3 (65.6% and 77.4%), and pT4 (3.1% and 0.0%) in the LATG and TLTG groups, respectively. The pN category: pN0 (50% and 38.7%), pN1 (21.8% and 29%), pN2 (18.8% and 25.8%), pN3 (9.4% and 6.5%) in the LATG and TLTG groups, respectively.

For postoperative complications and hospital stay duration (Table 11), the anastomotic leakage was an esophagojejunostomy-related complication, which occurred in 1 (3.1%) and 2 (6.5%) patients in the LATG and TLTG groups, respectively. The surgical wound infection occurred in LATG in 1 patient (3.1%).The mean postoperative hospital stays were 11.4 days and 10.77 days (*p = 0.6*) in the LATG and TLTG groups, respectively. There was no statistically significant difference between the two methods according to the hospital stay.

**Table 11:** Comparison of postoperative complications and hospital stay duration between LATG and TLTG groups.

Characteristic	LATG, *N* = 32^a^	TLTG, *N* = 31^a^	Overall, *N* = 63^b^	*p*-value^c^
catLNS				0.2
16 thru 30	28 (48%)	30 (52%)	58	
30 thru highest	3 (100%)	0 (0%)	3	
Lowest thru	1 (50%)	1 (50%)	2	

^a^ *n* (%).

^b^ *n*.

^c^ Fisher's exact test.

## 7 Discussion

The procedure that shows the greatest promise for enhancing patient outcomes is laparoscopic gastrectomy. The majority of reports had mentioned LATG, and esophagojejunostomy is performed with a circular stapler (12–14).

Although widely used, this extracorporeal technique has drawbacks, including the requirement for a mini-laparotomy and the difficulty of performing EJS within a constrained operating window (15). A highly skilled and experienced surgeon with experience performing total laparoscopic gastrectomy is required for the TLTG method, which uses endoscopic surgical devices to perform laparoscopic EJS. It is evident that a linear stapler is used to perform laparoscopic esophagojejunostomy intracorporeally.

Even with direct endoscopic vision, the EJS procedure can still be challenging, particularly for obese patients. Therefore, it is imperative to obtain a wide operating view (16). In the realm of laparoscopic gastrectomy, the most technically challenging kind of anastomosis is still EJS following LATG or TLTG.

Numerous studies comparing the surgical results of TLTG and LATG had been published; some of these studies compared the results of the procedures in the short term, while others assessed the safety and viability of extracorporeal and intracorporeal anastomosis (17–19). As a result, the primary goal of the current research in this area is to evaluate how tumor size and upper margin length affect survival rates.

We discovered that, out of the 63 patients who underwent TLG during the study period, 32 and 31 patients were in the LATG and TLTG groups, respectively. Therefore, following surgery, we observed that, of the LATG group, 25 (78%) were still alive, 6 (19%) had passed away, and 1 (3%) had experienced a recurrence. While 3 (9.7%) died, 1 (3.2%) experienced a recurrence, and 27 (87.1%) of the TLTG group were still alive. According to the multiple logistic regression analysis conducted on our data in the current study for assessing the connectivity and linkage between upper margin length and the tumor size on survival rate, we do not find any connectivity and risk-related of the upper margin length and tumor size with the survival rate, although the ratio tumor size/upper margin length do not have relationship with survival rate. Their respective *p*-value don't explain any impact on the survival (0.7, 0.2, and 0.08). However, the pathological tumor stage (pT), pathological nodes stage (pN), patients’ BMI and age have a connectivity and relationship with the survival rate.

According to Zhong X et al.'s study (19), regardless of the technique used during surgery, a multivariate cox regression analysis showed that the pathological LNs stage (*N*) was one of most important risk factors for laparoscopic surgery mortality and recurrence. In the few studies that have compared the TLTG and LATG, favorable and considerable long—term results have been demonstrated and reported, survival outcomes also revealed similar survival rates. In the long run, TLG do not raise the survival risk (19, 20). According to our current research, there is no statistically significant difference in the survival rate between the two laparoscopic gastrectomy techniques (TLTG, LATG).

According to Shen JG et al.'s (21) research, a presence of tumor's cells into proximal margin(positive margin) was linked to advanced and metastatic disease. The study also revealed that the tumor's size and the extent of its invasion were independent risk factors. According to the findings of his research, a positive margin in gastric cardiac adenocarcinoma is more indicative of advanced disease than it is of a standalone risk factor for survival. Even though a microscopic margin may have an impact on a poor prognosis in the early stages of a disease, it is no longer a reliable indicator of prognosis in later stages. Nonetheless, the pathological marker dictates the prognosis. Recurrence is not linked to proximal resection in cases of proximal gastric carcinoma (22, 23). As per the guidelines on western series by the Japanese Gastric Cancer Association (JGCA) (24), margin adequacy was found to be independently associated with Overall Survival in multivariate analysis. The distance of proximal margin resection is not a prognostic factor for patients who undergo curative total gastrectomy for advanced GC, according to Kim A (25). This is true even though many surgeons worldwide still follow the guidelines which lead them to obtain a safe resected proximal margin of 5–6 cm during advanced GC surgery. In comparison of LATG and TLTG groups; it was reported in these studies (26) that TLTG and LATG were not statistically and significantly different from outcomes, the blood loss was higher in LATG group than TLTG, surgery duration were longer in TLTG, they did not show differences for perioperative and postoperative complications.

Wei M et al. (19), reported that the results were better for TLTG group with linear stapler than the LATG with circular stapler. He revealed that the incidence of anastomotic stricture and stenosis, the blood loss were consistent with others results reported into these studies (27–29). Our study found the different results from those reported by some authors. The blood loss, the surgery duration were statistically and significantly different in TLTG group than LATG group, we have found the short time of surgery duration and lower blood loss in TLTG group than LATG group (*p = 0.006 and 0.005*) this can be related to the assistant incision made in the LATG which can increase the amount of blood loss, therefore the anastomosis performed through the narrow incision, and the hand-sewn reinforcement of anastomosis can also increase the time of surgery. Our results are comparable with results reported by Chen K, Mou YP et al. (30), intraoperative blood loss was lower in TLTG than LATG groups but the resected LNs’ number and hospital stay duration were considerable better in the in the TLTG than LATG group.We didn't find significant difference into distance of mobilized esophagus, intraoperative complications, previous history of surgery and tumor size. The number of LNs dissected and positive lymph nodes.

About postoperative complications, we have found in the current study that the anastomotic leakage represent the esophagojejunostomy-related complication. It occurred in 1 (3.1%) and 2 (6.5%) patients in the laparoscopic assisted total gastrectomy (LATG) and total laparoscopic total gastrectomy(TLTG) group, respectively. The surgical wound infection occurred in LATG to 1 patient (3.1%). No significant and statistically difference in the two groups. The hospital stay length is similar into the two methods.

The esophagoejunostomy can be linked with fatal complication such as stricture and bleeding. Bleeding mostly complicate the prognosis of patient and lead to death in severe cases. It has been showed that anastomosis leakage can finally affect the survival rate of patients with advanced GC (31). According to Park K et al. (32) reports the high incidence of anastomotic leakage into circular stapler group was reported compared with linear stapler group. These complications can prolong length the hospital stay, in some cases they can be associated with the overweight, age and comorbidities which can be a patent risk factor to the outcome (33).

Ito H et al. (34) reported in his study that the absence of lymph nodes metastasis and RO resection emerged as factor independent associated with improved postoperative survival. The frequency with which proximal resection margin was infiltrated with cancer was function of gross margin length and Stage. Proximal length of at least 6 cm was required to achieve a microscopically negative proximal margin for T3 and T4 cancer.

In our study, the means upper margin length were 3 cm (2–3) and 2 cm (2–3) (*p = 0.052*) in LATG and TLTG group, respectively. Frequencies of pT3 (65.6% and 77.4%), pN3 (9.4% and 6.5%) in the LATG and TLTG group, respectively.

As the clearance of nodal micrometastasis is increased from a retrieval of a great number of LNs in GC, this can improve staging accuracy and patients’ survival of (35, 36). The status of nodes has been considered like one of significant prognostic factors in GC. According to Seevaratnam R et al.'sguidelines (37), a greater number of LNs should be evaluated, with a recommendation to assess 16 LNs, particularly in cases of advanced GC. According to Liu YY, Fang WL et al. (36–38) retrieving more than 25 lymph nodes during curative-intent gastrectomy substantially improved survival and survival stratification of advanced gastric cancer without compromising patient safety.The lymph nodes sampling was adequate in the two groups because the number of resected and assessed lymph nodes exceed 16 LNs in TLTG and LATG. The number of resected lymph nodes was similar no difference found between the two methods, the mean were 19.50 (18–22) in LATG and 22 (19–25).

## 8 Conclusion

The current study showed that upper margin length and tumor size do not have a relationship with survival rate. They are not independent risk factors for the outcome and survival rate of the compared esophagojejunostomy (EJS) methods for gastric cardia cancer treatment. We can also conclude that EJS using a linear stapler requires a shorter surgery duration and less blood loss than EJS using a circular stapler, while having similar anastomotic-related complications, hospital stay length, and survival rate.

## Data Availability

The raw data supporting the conclusions of this article will be made available by the authors, without undue reservation.
